# Peripheral Neuropathy in Sézary Syndrome: Coincidence or a Part of the Syndrome?

**DOI:** 10.4274/Tjh.2012.0163

**Published:** 2013-12-05

**Authors:** Yeşim S. Karadağ, Aydın Gülünay, Neşe Öztekin, Fikri Ak, Saadettin Kılıçkap

**Affiliations:** 1 Ankara Numune Research and Training Hospital, Department of Neurology, Ankara, Turkey; 2 Hacettepe Cancer Institute, Department of Preventive Oncology, Ankara, Turkey

**Keywords:** Sézary syndrome, mycosis fungoides, Axonal neuropathy

## TO THE EDITOR

**Figure 1:**
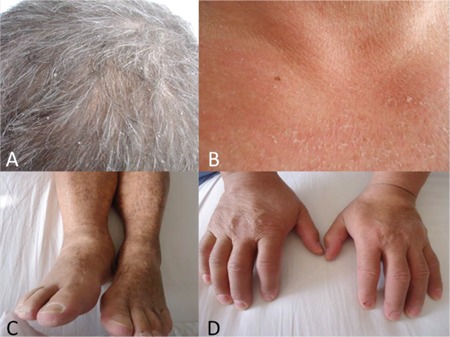
Widespread squames on the scalp and trunk (A and B), and a diffuse dryness with widespread squames on the arms and thighs (C and D).

A 48-year-old male patient was admitted with the complaints of diffuse skin dryness, fatigue, and numbness of the hands. He reported cramps and fasciculation occurring at nights and weakness of the arms. His physical examination (AG) revealed a diffuse dryness with widespread squames on the scalp, trunk, arms, and thighs (Figure 1). Neurological examination revealed hypoactive deep tendon reflexes in both lower extremities and hypoesthesia of gloves-socks type. His white blood cell count was 43,600/mm^3^, 50%-55% of which comprised eosinophils. Flow cytometry revealed a Sézary cell count of 3200/µL, a CD4/CD8 ratio greater than 22.5, and less than 7% T-cells that expressed CD7. The same clonal amplification was demonstrated by T-cell receptor gene analysis of skin, blood, and lymph nodes. Computerized tomography showed axillary and inguinal lymphadenopathies. The patient was at stage IIA.

Biopsies from skin lesions and lymph nodes were performed. Immunohistochemical examination was consistent with SS, showing diffuse CD4 staining. Diagnosis of motor axonal neuropathy was based on the decrease of compound muscle action potential amplitudes of right median, peroneal, and bilateral posterior tibial nerves in a nerve conduction study. Other etiologies for motor axonal neuropathy such as diabetes mellitus were excluded. PUVA therapy was used for his skin lesions. Photopheresis was performed once a month. After the therapy, his neuropathy improved. 

## DISCUSSION

Polyneuropathy in patients with cancer may be related to chemotherapy and is rarely due to direct invasion of the nerve [6,7]. Neuronal antigens expressed by the tumor stimulate an immune response characterized by T cells, antibodies, or both, which attack not only the tumor but also the neural tissue [8]. Although these scenarios could be expected in lymphomas, nervous system involvement is not commonly reported in reviews of SS [1,2]. 

The co-occurrence of SS and peripheral neuropathy was first reported in 1978 by Bargman and Coupe [5]. Another case of SS with neurolymphomatosis was reported by Bezier et al. [3]. In that case, muscle and nerve biopsy specimens showed neurogenic muscle atrophy and an axonal neuropathy secondary to an epineurial and endoneurial infiltration by Sézary cells [3]. Although extracutaneous spread is not uncommon in advanced stages of SS, neurological complications are rare and result from leptomeningeal or central nervous system involvement. 

Our patient had axonal peripheral neuropathy without central nervous system involvement. Neuropathic symptoms started prior to his medications, which reassured us that the responsible etiology may have been his primary disease. The neuropathic symptoms of the patient appeared at the time of diagnosis and improved after treatment. These findings support the idea that the neuropathy associates with SS and may be a consequence of paraneoplastic syndrome. 

This case emphasizes the importance of keeping polyneuropathy in mind while dealing with patients with SS, although it is difficult to treat, and suggests the possible role of neurotropism of malignant cells. 

## CONFLICT OF INTEREST STATEMENT

The authors of this paper have no conflicts of interest, including specific financial interests, relationships, and/ or affiliations relevant to the subject matter or materials included.
